# Exogenous Application of Cellulose-Based Materials for Improved Plant Fitness

**DOI:** 10.3390/ijms27177629

**Published:** 2026-08-26

**Authors:** Tatiana Komarova, Kamila Kamarova, Michael Taliansky

**Affiliations:** 1 Shemyakin-Ovchinnikov Institute of Bioorganic Chemistry, Russian Academy of Sciences, 117997 Moscow, Russia; michael.taliansky@mail.ru; 2 Belozersky Institute of Physico-Chemical Biology, Lomonosov Moscow State University, 119991 Moscow, Russia; 3 Vavilov Institute of General Genetics, Russian Academy of Sciences, 119333 Moscow, Russia; kamila.kamarova@yandex.ru

**Keywords:** cellulose, nanocellulose, coating, biotic stress, abiotic stress, cellulose nanocrystals, cellulose nanofibers, plant immunity priming, elicitor

## Abstract

Exogenous application of bio-based nanomaterials provides a targeted strategy to modulate plant physiological and biochemical responses. This review synthesizes recent advancements in the foliar application of nanocellulose (NC), in particular, cellulose nanocrystals (CNC) and cellulose nanofibers (CNF), to enhance plant fitness. CNC-formed films provide physical and biochemical barriers that increase plant drought and cold stress tolerance. Topically applied CNC reduce non-stomatal transpiration and serve as insulators, allowing the flowering buds to successfully survive chilling, avoid freezing, and maintain cell membrane integrity. Simultaneously, CNC- and CNF-formed coatings are porous enough not to block the natural gas exchange essential for plants. CNC trigger internal antioxidant defense systems, upregulating reactive oxygen species-scavenging enzymes and modulating molecular signaling cascades. NC foliar treatment suppresses the growth of pathogenic bacteria and fungi, interferes with their adhesion and plant tissue penetration, and prevents biofilm formation. Thus, topical NC application could be regarded as a multi-functional tool for precision crop management and protection.

## 1. Introduction

**Figure 1:**
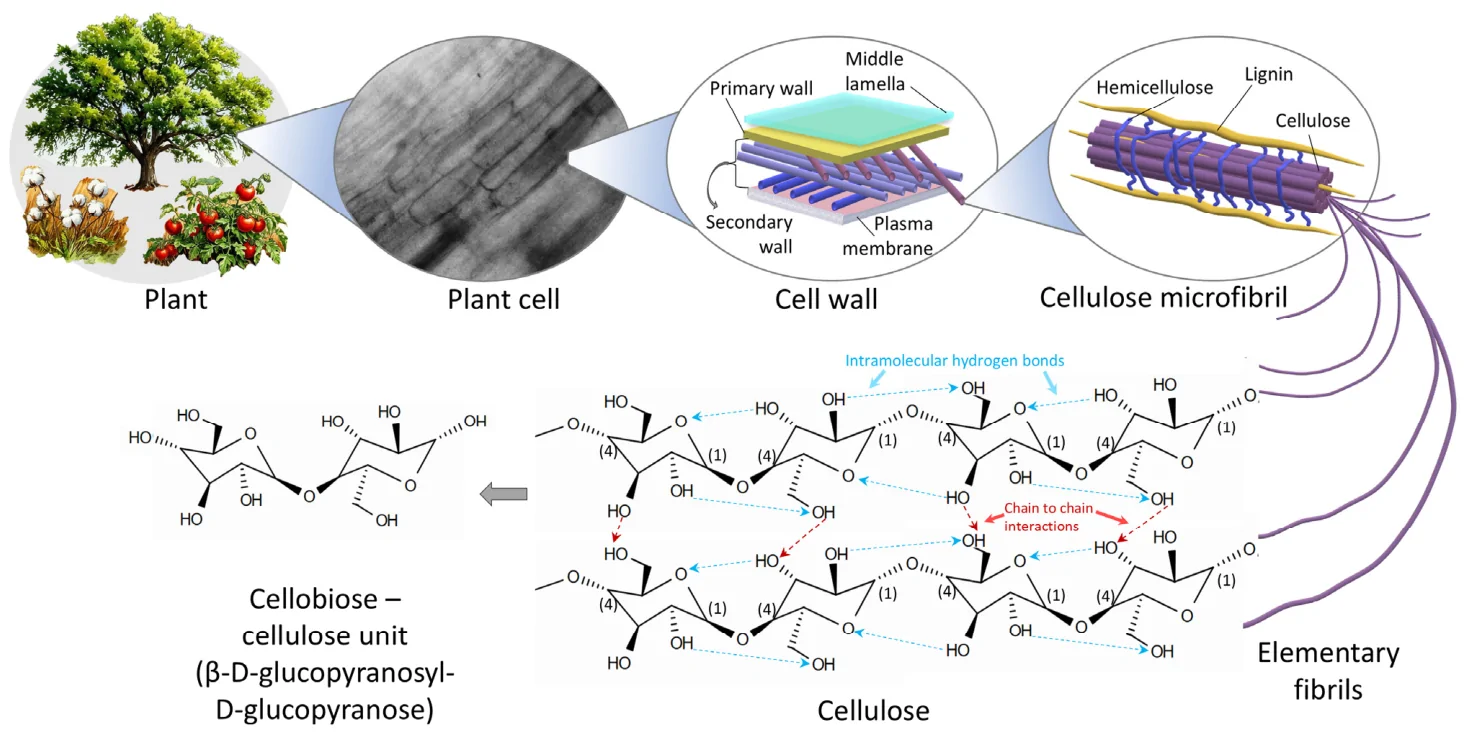
Schematic representation of the plant cell wall and cellulose fibril hierarchy. Intermolecular (chain-to-chain) hydrogen bonds are indicated by red dashed arrows, while intramolecular hydrogen bonds are denoted by blue arrows. Adapted from Casanova et al. [9]; used under the Creative Commons Attribution 4.0 International License (CC BY 4.0).

Plants are the major source of cellulose, which comprises about half of plant biomass. Cellulose is the main structural component and functional scaffold of the plant cell wall (CW), providing its mechanical rigidity, defining cellular morphology, and resisting internal turgor pressure to maintain structural integrity during growth and development. Cellulose fibrils are embedded in the CW matrix, composed of hemicelluloses and pectins [1,2,3]. Cellulose molecules are synthesized by plasma membrane-localized multimeric cellulose synthase complexes that form hexameric rosettes [4], while CW polysaccharides and pectins are produced in the Golgi apparatus and transported to the CW [5]. Cellulose consists of β-D-glucopyranosyl monomers linked by (1→4) glycosidic bonds (Figure 1). Thus, cellulose is actually formed from cellobiose units inverted relative to each other. This leads to the formation of regular ribbon-like molecules that associate into microfibrils due to inter- and intramolecular hydrogen bonds and van der Waals forces [6]. The structure of cellulose synthetase complex rosettes defines the initial assembly of individual molecules into elementary fibrils (composed of three molecules) and into the higher-order microfibrils of 18 strands, which are believed to be organized in six or five layers comprising 2-3-4-4-3-2 or 3-4-4-4-3 molecules, respectively [7,8] (Figure 1). This model is consistent with evidence obtained using numerous structural analysis methods (e.g., high-resolution microscopy, NMR, X-ray or neutron scattering). Despite the prefix “micro-“ the dimensions of cellulose microfibrils are nanoscopic: the diameter is usually estimated at 2.5–4 nm and does not exceed 5 nm [8]. The hydrogen bond network determines the presence of crystalline and amorphous regions in these cellulose microfibrils (Figure 1) as well as water insolubility.

Depending on the plant species, developmental stage, type of tissue, external stimuli, etc., CW compositions could significantly vary. The secondary CW of most vascular plants, especially woody plants, contains lignin, a heterogeneous aromatic polymer [10,11], which is the second (after cellulose) most abundant natural polymer on the planet. Lignin is deposited in the CW of particular plant tissues, especially vasculature. It provides structural and mechanical support and plays a significant role in plant defense against pathogens [12]. Multiple cellulose microfibrils associated with lignin and hemicelluloses form stable macrofibrils in the CW.

Cellulose is mainly obtained from woody and herbaceous plants or agricultural waste, though other cellulose sources include algae and tunicates. Moreover, cellulose can be synthesized by bacteria [13,14,15]. Plant-derived cellulose is produced by chemically and/or mechanically separating cellulose fibrils from the CW matrix, lignin and other components. This extraction process involves the simultaneous or sequential disintegration of cellulose macro- and microfibrils. The method for cellulose purification depends on the purpose and desirable features of the final product, as various approaches result in cellulose with different properties (e.g., fiber length, purity, homogeneity, etc.). Tightly packed crystalline regions of cellulose microfibrils are hard to break; thus, during purification and extraction processes, the microfibrils are disrupted in their looser amorphous regions. Cellulose production from plant biomass requires the removal of lignin through energy-consuming and chemical-intensive processes that are both costly and environmentally hazardous [10,16]. However, milder and eco-friendlier methods and techniques based on cellulose extraction with ionic liquids and deep eutectic solvents are currently being developed. The combination of low-cost feedstocks (agricultural postharvest and pruning waste) [17,18] with “green” pretreatment methods (steam explosion, usage of deep eutectic solvents, autohydrolysis) [19,20,21] offers the best balance of economic feasibility and environmental sustainability for agricultural applications. Cellulose has a long history of manufacturing and utilization, primarily in the paper and textile industries, which use cellulose fibers [22,23]. Today, packaging production and various biomedical, agricultural and environmental applications have significantly widened this area [24,25,26,27]. The novel wave of interest in cellulose and its derivatives was raised with the emergence of nanocellulose (NC), whose properties are markedly different even though the initial compound is the same [15,28,29]. NC possesses the core characteristics of native cellulose, including biocompatibility, biodegradability, relatively low feedstock cost, and low density. Simultaneously, it introduces novel, tailorable features such as variable particle or fiber dimensions, diverse morphologies, crystallinity, tunable optical properties, and enhanced tensile strength, which are determined by the source and production method. Due to its biocompatibility and biodegradability, this nanomaterial offers promising perspectives for various agricultural applications, ranging from protecting crops and seeds against adverse environmental conditions and pathogenic microorganisms to serving as a carrier for the delivery and controlled release of nutrients, pesticides, and fertilizers. There are two main classes of plant-derived NC: cellulose nanocrystals (CNC) and cellulose nanofibers (CNF) [30]. CNC are obtained from cellulose crystalline regions and are represented by rod-shaped nanoparticles of different sizes, varying from 5 to 70 nm in width and 100–300 nm in length, depending on the source from which they were extracted. CNCs are characterized by high crystallinity, mechanical strength and stiffness [30,31]. Under certain conditions, CNC could form a film with low thermal conductivity [32]. CNF originate from amorphous and crystalline cellulose regions and are represented by fibers with micrometer-scale lengths and widths of 5–60 nm. This type of NC is characterized by flexibility and a high film-forming capacity [15,30]. Both CNC and CNF demonstrate water-holding ability, making them excellent components of superabsorbent gels and water-retention coatings [33]. Films formed by either CNC or CNF reduce surface tension and enhance leaf wettability. Moreover, CNF obtained by the aqueous counter collision method [34] exhibit amphiphilic properties [35]. The adjustable porosity of NC films allows for the maintenance of normal gas exchange in plant tissues after treatment with NC-based formulations [15]. Due to NC’s rain-fastness and ability to adhere to plant leaves, its application in combination with agrochemicals prolongs the effect of active compounds [36,37].

Over the last decade, numerous excellent reviews on the extraction of plant-derived cellulose and NC and their agricultural applications have emerged. Most focus on approaches to NC extraction and the potential use of NC and its derivatives for the delivery of traditional agrochemicals or “green” pesticides, such as chitosan, tannic and gallic acids, and biocontrol microorganisms (e.g., [15,16,17,27,28,29,38,39,40]). Indeed, the major areas of NC utilization in agriculture discussed in recent literature reviews include (1) the “packaging” and controlled release of various pesticides, nutrients and biostimulants [39,40] and (2) soil structural improvement and water retention as a hydrogel component [33,41,42,43]. However, insufficient attention has been paid to the intrinsic NC features that underlie its influence on plant growth, development and stress resilience; thus, the effects of NC per se on plants have not yet been summarized. The current review focuses on the physical and molecular impacts of foliar-applied NC, specifically CNC and CNF, on plant stress signaling and the suppression of phyllosphere pathogens while excluding the abovementioned areas of NC application.

## 2. Abiotic Stresses

### 2.1. Cold Stress

Cold stress severely hinders plant development, growth, and survival due to its negative effect on cellular biochemical reactions and significant decrease in nutrient and water uptake. Moreover, chilling induces stomatal closure and suppresses photosynthesis and transpiration. The response to low temperatures includes both slowed metabolism due to suboptimal conditions for enzyme function and numerous transcriptional, post-transcriptional and post-translational changes [44,45,46]. In addition, chilling and especially freezing can cause damage to cell membranes, increase membrane permeability, lead to electron leakage, and induce the production of reactive oxygen species (ROS) [47]. Spring frosts cause severe cold damage to the reproductive buds of stone fruit trees and grapevines, leading to substantial crop losses. To prevent the adverse effects of cold stress and to protect plants from freezing, various approaches are used, including wind machines, heaters, sprinkler irrigation and polypropylene or polyethylene row covers [48,49]. However, there is a need for novel techniques and materials for the preservation of vulnerable buds and developing flowers of the fruit trees and vines.

A CNC suspension forms a film upon slow water evaporation, and the thickness of the film depends on the concentration of the initial suspension. Such CNC films are characterized by low thermal conductivity (0.061 W m^−1^ K^−1^) [32]; thus, they can act as thermal barriers since materials with thermal conductivities of ca. 0.2 W m^−1^ K^−1^ at room temperature are commonly considered thermal barriers or insulators [50,51]. This CNC feature has been successfully exploited to protect the developing buds of sweet cherry, apple, and peach, as well as dormant grape buds, from freezing [32,52,53]. A 2% or 3% CNC suspension [54] applied to buds via electrostatic spraying was shown to improve their tolerance to low temperatures by about 2–4 °C when the treatment was performed 24 h prior to freezing. Cold hardiness increased with time, i.e., on the 1st day after treatment, the ice nucleation temperature was 2.5 °C lower compared to the control, and by the 3rd day, it was 5.5 °C lower. Moreover, increasing the CNC concentration had a disproportionate effect: while a 1% suspension did not enhance cold hardiness, a 2% suspension had a significant effect, and the use of a 3% suspension resulted in much more prominent protection of the buds. Lee et al. [53] observed that CNC treatment of peach flowering buds reduced pistil damage after a −4 °C cold challenge. This is a critical finding because pistil integrity is essential for fruit formation, whereas stamen damage can often be overcome by artificial pollination. Details of the experimental design and the effects of NC treatment are summarized in Table 1. Notably, in all the abovementioned studies, no negative effects on floral development or fruit set were observed. These data establish CNC dispersions as sprayable frost protectants for fruit crops through purely physical effects: CNC films function as both an insulating barrier and an ice nucleation inhibitor [52,53]. However, it cannot be excluded that CNC also induce specific biochemical and/or transcriptomic changes in plant tissues that underlie increased cold tolerance; thus, further studies could reveal the molecular basis of CNC-mediated protection from cold.

### 2.2. Drought Stress

Drought stress significantly impairs plant productivity and biomass accumulation due to specific physiological and biochemical changes in plant tissues [55,56]. Cell division and elongation are inhibited under water-deficit conditions [57]. Drought increases oxidative stress via excessive ROS generation, leading to potential damage to major biomolecule classes such as lipids, proteins, carbohydrates, and nucleic acids [58]. In addition, water deficit decreases stomatal conductance and transpiration, negatively affects photosynthesis and reduces carotenoid and chlorophyll contents [59]. Notably, there is a significant overlap between sets of drought- and cold-responsive genes and the general molecular pathways involved in plant adaptation to these stresses [60].

Due to its film-forming and water-retention abilities, NC could be regarded as a promising material for improving plant fitness under drought stress [39,61]. There are numerous examples of cellulose as a component of hydrogels that are used for soil improvement and water retention [33,62]. However, it is not only the physical properties of cellulose and NC that are beneficial for agricultural purposes. In contrast to the aforementioned examples of cold tolerance induction, CNC have both physical and biological effects on plant drought tolerance, as demonstrated by Li et al. [63] in tall fescue, an important forage and turf-forming grass widely used for ecological restoration of slopes [64]. Foliar spraying of tall fescue with a 0.01% CNC suspension stimulated plant growth and increased chlorophyll content and the photosynthetic rate under normal conditions (Table 1). Notably, Li et al. [63] used 100-fold lower CNC concentrations than in the abovementioned studies by Alhamid et al., Lee et al., and Arnoldussen et al. [32,52,53]. Under drought stress, CNC-treated plants demonstrated better adaptation and overall performance compared to untreated control plants. CNC were shown to affect gene expression, resulting in better plant fitness; they alleviated the negative effects of drought stress by reducing ROS bursts, enhancing the activity of the antioxidant enzymes, and increasing the levels of ROS scavengers (e.g., proline) [63].

Collectively, these findings demonstrate that CNC confer protection against diverse abiotic stresses by acting both as a physical shield and a molecular modulator that enhances physiological fitness and reduces oxidative damage.

**Table 1:** Examples of exogenous NC application for plant stress resilience.

Stress/Pathogen	Cellulose Type	Plant	Outcome	Application	References
			Abiotic stress		
Cold	CNC, commercial source	Sweet cherry (*Prunus avium* L.), grape (*Vitis vinifera*), apple (*Malus domestica* L.)	Improved cold-hardiness of sweet cherry, apple and grape buds by about 2–4 °C compared to nontreated buds	A 2–3% (all % concentrations correspond to *w*/*v* unless otherwise specified) CNC suspension with cetrimonium bromide surfactant and a trace amount of stabilizing agents (patented formulation [54]) was applied to buds via a single-nozzle electrostatic sprayer (68.95–96.53 kPa); buds were treated in the field and collected 24 h after treatment for cold stress assays	[32,52]
	CNC, source is not specified	Peach (*Prunus persica* L.)	Improved cold-hardiness of peach flowering buds compared to nontreated buds upon −4 °C cold challenge, while no significant effect was observed at −6 °C; notably, overall flower bud damage and in particular stamen damage was lower in the CNC-treated group	Two-year-old “Daewol” peach trees commercially grafted on wild peach seedlings, planted in pots; the trees were used after the chilling requirement was met in a natural condition; the plants were sprayed with 2% CNC suspension and dried completely in the field for more than 12 h then transferred to Soil–Fruit–Daylit–System (SFDS) chambers	[53]
Drought	CNC, commercial source	Tall fescue (*Festuca arundinacea*)	(i) Increased plant height, leaf length, chlorophyll *b* content, and photosynthetic rate; (ii) alleviated drought-induced growth inhibition and the associated decrease in chlorophyll *b* content, photosynthesis, transpiration rate, and stomatal conductance; (iii) upregulated expression of genes associated with photosynthesis and genes involved in strengthening and stabilizing the plant cell wall	A 0.01% CNC suspension supplemented with 0.05% Tween-20 was sprayed on leaves every three days for 20 days under greenhouse conditions	[63]
			Biotic stress		
*Pseudomonas syringae* pv. *tomato*	CNC (from commercial microcrystalline cellulose), CNC-PLGA NPs (poly(DL-lactide-co-glycolide acid) (PLGA) copolymer-based biopolymeric nanoparticles (NPs), CNC used as stabilizer), CNC from tomato waste (in-house preparation)	Tomato (*Solanum lycopersicum*)	CNC and CNC-PLGA treatments enhanced tomato plant growth, with their height exceeding the control by 10–15% at 14 days after treatment; 0.1% CNC application resulted in increased mean leaf area; CNC reduced *P. syringae* pv. *tomato* load, while CNC-PLGA NPs completely eliminated the pathogen, yielding zero detectable bacteria	Suspensions of 0.5% CNC, 0.1% CNC, CNC-PLGA NPs, or 1% CNC were sprayed on tomato plants cultivated in a growth chamber; for in vivo experiments, the plants were spray-inoculated with a bacterial suspension 24 h after treatment	[65,66,67]
*Pseudomonas syringae* pv. *tomato*	CNC, commercial source	*Arabidopsis thaliana*	(i) Bacterial content in infiltrated leaves was reduced by more than 2-fold after 0.025% or 0.05% CNC treatment; (ii) transcriptomic analysis revealed upregulation of genes associated with immune reactions and bacterial/fungal infection, as well as genes involved in CW remodeling in response to 0.05% CNC treatment; (iii) CNC minimally interfered with seedlings’ growth: treatment with 0.0125% or 0.05% CNC treatment reduced growth by 7 and 11%, respectively, relative to mock-treated plants	The leaves of plants cultivated in a growth chamber were infiltrated with a 0.025% or 0.05% CNC dispersion and 24 h later infiltrated with a *P. syringae* suspension (OD_600_~0.002 that roughly corresponds to 2 × 10^6^ CFU mL^−1^); samples were collected 3 days after bacterial challenge; of note, leaf infiltration with 0.075% CNC had negative effect on plant; for transcriptomic study, 14-day-old seedlings were treated with liquid media supplemented with 0.0125% or 0.05% CNC; samples were collected 1 h after treatment	[68]
*Pseudomonas syringae* pv. *savastanoi*	CNC from olive pruning waste (in-house preparation)	Olive tree (*Olea europea*)	In vitro: (i) inhibition of bacterial growth by 1% CNC (81.6%) was comparable to that of 0.3% copper sulphate (89.7%), (ii) a wide range of CNC concentrations from 0.05 to 1% significantly reduced bacterial biofilm formation after 12 h of incubation; in vivo: a 1% CNC suspension restricted bacterial growth	Foliar treatment of 1-year-old seedlings with a 1% CNC suspension; bacterial inoculation was performed 24 h later by spraying plants with a bacterial suspension (1 × 10^6^ CFU mL^−1^)	[69]
*Pseudomonas cannabina* pv. *alisalensis*	CNF (Nanoforest^®^), commercial source	Cabbage (*Brassica oleracea* var. *capitata*)	CNF foliar treatment reduced *P. cannabina* pv. *alisalensis* disease symptoms and epiphytic populations by preventing bacterial entry; CNF coating altered leaf surface hydrophobicity, decreased flagellar motility, and downregulated key bacterial virulence genes, including those for type III effectors, coronatine biosynthesis, and flagellin	A 0.1% (*v*/*v*) CNF suspension in water supplemented with 0.025% Tween 20 was sprayed on leaves of 2-week-old plants cultivated in a growth chamber	[70]
*Xanthomonas arboricola* pv. *corylina*	CNC from hazelnut lignocellulosic waste (in-house preparation)	Hazelnut (*Corylus avellana*)	In vitro: bacterial growth inhibition at levels comparable to that of 0.6% copper oxychloride (a common field pesticide); in vivo: CNC foliar application led to a significant reduction in disease incidence to levels comparable to those in plants sprayed with copper oxychloride; the severity of symptoms was reduced in CNC-treated plants compared to a water-treated negative control	Leaves of micropropagated 1-year-old seedlings placed in a growth chamber were treated with a 1% CNC suspension to obtain a uniform coating; 24 h later, the plants were spray-inoculated with a bacterial suspension (1 × 10^6^ CFU mL^−1^)	[71]
*Phakopsora pachyrhizi*	CNF (Nanoforest^®^), commercial source	Soybean (*Glycine max* cv. Enrei)	Surface properties of CNF-sprayed leaves were converted from hydrophobic to hydrophilic, resulting in suppression of genes related to chitin synthesis involved in the formation of pre-infection structures; CNF treatment led to suppression of pre-infection structure (germ tube and appressoria) formation and reduction in lesion area and number	A 0.1% (*v*/*v*) CNF suspension in water supplemented with 0.025% Tween 20 was sprayed on leaves of 3-week-old soybean plants; after drying at room temperature for 3–4 h, the leaves were spray-inoculated with a suspension of urediniospores containing 1 × 10^5^ spores mL^−1^; lesions were counted on the 10th day of infection	[72]
*Helminthosporium solani*	CNF (Nanoforest^®^) from bamboo or softwood, commercial source	Potato (*Solanum tuberosum*)	Fungal growth was significantly suppressed: more than 2-fold reduction in *H. solani* DNA levels and halved amount of conidia produced per gram of minituber compared to control were observed; the inhibiting effect of CNF on *H. solani* sporulation in trials on microtubers was from strong to moderate depending on the trial and potato cultivar	In vitro cultivated microtubers were incubated with a 0.1% CNF suspension, air-dried and inoculated with *H. solani* conidia; hydroponically grown minitubers were treated with a 0.1% suspension of CNF supplemented with 0.01% (*v*/*v*) Silwet L-77 using an airbrush sprayer, air-dried and inoculated with an *H. solani* conidia suspension (1 × 10^−5^ conidia mL^−1^); *H. solani* propagation was assessed 2 (for microtubers) or 5 (for minitubers) weeks after inoculation	[73]

## 3. Biotic Stress

### 3.1. Bacterial Pathogens

*Pseudomonas syringae* is a pathogenic Gram-negative bacterium that causes significant damage to a wide range of cultivated agricultural plants [74,75]. It is an extracellular pathogen that enters plant tissues through wounds or stomata. Infection occurs in two stages: an epiphytic phase on the plant surface, followed by an endophytic phase colonizing the intercellular apoplastic space [75,76].

Several studies by Giorgio Balestra’s research group have demonstrated that CNC and CNC-based formulations are effective against *P. syringae* by restricting its growth, inhibiting biofilm formation, and eliminating infection. Fortunati et al. obtained poly(DL-lactide-co-glycolide acid) (PLGA) nanoparticles (NPs) using CNC as a natural stabilizer (CNC-PLGA) and demonstrated that CNC-PLGA NPs had a bactericide effect on *P. syringae* pv. *tomato* (*Pst*), while CNC alone exerted a bacteriostatic effect [65]. In addition, both CNC and CNC-PLGA NPs had no phytotoxic effects when applied exogenously to tomato plants; moreover, such treatment even slightly stimulated plant growth. Similar results were obtained by Schiavi et al. in the tomato-*Pst* pathosystem: it was demonstrated that CNC obtained from tomato postharvest waste are effective in vitro against *Pst* and could be used to reduce epiphytic populations of *Pst* [66]. Another study by Schiavi et al. [69] showed that CNC obtained from olive pruning waste could be regarded as an agent for *P. syringae* control. The efficiency of that formulation was evaluated against the causative agent of olive knot disease, *P. syringae* pv. *savastanoi* (*Psav*). A 1% CNC suspension suppressed *Psav* growth and biofilm formation in vitro. Moreover, similar to the abovementioned results on *Pst* and tomato plants [65,66], CNC restricted the multiplication of epiphytic bacteria on treated olive leaves [69]. *Pst* propagation on CNC-treated leaves was significantly reduced and comparable to that on copper-treated leaves at 7 days after application. However, by the 14th day, *Pst* levels in the CNC-treated group were indistinguishable from those in the water-treated control, indicating the necessity for repeated applications. Notably, the in vitro antibacterial properties of different CNC preparations vary significantly [66,67,69], highlighting the dependence of their physicochemical characteristics and biological activity on both the source and the specific protocol for CNC extraction.

The antibacterial properties of another NC type—CNF—were examined on cabbage plants challenged with *P. cannabina* pv. *alisalensis* (*Pcal*), which induces bacterial blight and causes severe economic losses. This strain is resistant to traditional copper-based chemicals used for *Pseudomonas* control; thus, alternative approaches are in high demand. It was demonstrated that spray-coating cabbage leaves with CNF led to the restriction of *Pcal* infection [70]. A key feature of the CNF used in this study is their amphipathic nature, which allows them to reverse surface wettability, i.e., to convert hydrophilic surfaces to hydrophobic, and vice versa. Due to this property, the CNF coating made the leaves more hydrophilic, which resulted in reduced bacterial motility, thereby decreasing the incidence of *Pcal* entry. Furthermore, infiltration assays demonstrated that CNF only provide protection prior to *Pcal* penetration into plant tissue, as no effect was observed when the bacteria were directly injected into the leaves [70].

Another pathogenic bacterium that was shown to be successfully controlled using CNC-based materials is *Xanthomonas arboricola* pv. *corylina* (*Xac*). This Gram-negative bacterium causes serious damage to hazelnut plants, especially young ones, by inducing bacterial blight disease. In a study by Schiavi et al. [71], CNC variants obtained from hazelnut shells or hazelnut pruning waste were shown to be effective antibacterial agents, suppressing bacterial growth in vitro and in vivo. Hazelnut seedlings treated with a 1% CNC suspension demonstrated higher resistance to *Xac* infection: the proportion of the symptomatic plants was lower than that of water-treated plants; the efficacy of CNC treatment appeared to be comparable to that of copper oxychloride, a common antibacterial pesticide, demonstrating the high potential of CNC as an eco-friendly alternative to traditional chemical pesticides.

CNC were also used as a carrier component of composite nanostructured microparticles with chitosan chloride as an active antibacterial component to control *P. syringae* pv. *tomato* and bacterial speck disease development in tomato plants [77].

Notably, all aforementioned CNC-based formulations tested against *P. syringae* and *X. arboricola* were shown to have no adverse effects on plant growth, development, and vital biochemical parameters. On the contrary, Squire et al. [68] observed slight (7 or 11%) growth inhibition of *Arabidopsis thaliana* seedlings cultivated on a medium containing 0.025 or 0.05% CNC. In this experimental design, although the CNC concentrations were lower than in the aforementioned studies, the treatment was more intensive, involving continuous root uptake over 7 days in contrast to a single-dose foliar spray [68], which could explain the moderate negative effect on plant growth.

These examples demonstrate the possibility of utilizing natural cellulose-derived formulations to control bacterial diseases in agricultural plants. In addition to reducing the amount of chemical pesticides used, this approach is based on the valorization of “green” waste that accumulates during cultivation of the same plants that are to be protected from pathogenic bacteria—methods of CNC production from tomato postharvest residues, hazelnut waste and olive pruning material were developed [67,69,71]. Such studies pave the way toward “circular” supply chains and environmentally friendly approaches for sustainable agriculture.

### 3.2. Fungal Pathogens

*Phakopsora pachyrhizi* is an obligate biotrophic fungal pathogen and the causative agent of Asian soybean rust, a disease leading to serious soybean losses worldwide. *P. pachyrhizi* is transmitted by wind, which transfers its specialized spores—urediniospores. These spores do not require open stomata to penetrate plant tissues as they are capable of forming pre-infection structures—germ tubes and appressoria—allowing them to colonize epidermal cells [78]. Saito et al. reported that treating soybean leaves with a 0.1% suspension of amphiphilic CNF 4 h prior to inoculation with *P. pachyrhizi* urediniospores resulted in reduced lesion size and number compared to control leaves, indicating that CNF application enhanced the resistance of the soybean plants to this fungal pathogen [72]. The protective effect resulted from a marked decrease in the formation of pre-infection structures, which are essential for colonizing plant tissues. Transcriptomic analysis of *P. pachyrhizi* propagated on CNF-treated leaves revealed significant gene expression changes; specifically, the downregulation of genes involved in chitin synthesis led to the impaired formation of pre-infection structures. The authors attributed the reduced infection efficiency of *P. pachyrhizi* to CNF-mediated alterations in leaf surface hydrophobicity: coating the leaves with amphiphilic CNF made the surface hydrophilic, thereby preventing effective colonization by the pathogen [72].

CNF were successfully used to suppress the propagation of another fungal pathogen, *Helminthosporium solani*, a slow-growing ascomycete that infects potato tubers and causes silver scurf, a disease that results in “cosmetic” defects on the tuber, leads to weight loss during storage, and negatively affects the appearance of fresh-market tubers [79]. The emergence of fungicide-resistant isolates dictates the need for alternative sustainable methods for *H. solani* control. Moroz et al. reported that the treatment of potato micro- and minitubers with an amphiphilic CNF suspension reduced *H. solani* sporulation and propagation [73]. Although this study focuses on tuber surface application rather than foliar spraying, it highlights recent advancements in the field and confirms the suitability and practical utility of CNF-based treatments for the control of fungal pathogens from different taxonomic groups.

Taken together, these findings demonstrate that NC-based materials effectively counteract biotic stresses, with CNC being predominantly shown to protect against bacterial pathogens, while CNF exhibit efficacy against both bacteria and fungi. By serving as a physical barrier and modulating pathogen motility, adhesion, and gene expression, NC significantly reduces the ability of these organisms to penetrate and colonize plant tissues. A more detailed discussion of the specific mechanisms underlying this NC-mediated protection is provided in the following section.

## 4. Putative Mechanisms of NC-Induced Plant Stress Adaptation

**Figure 2:**
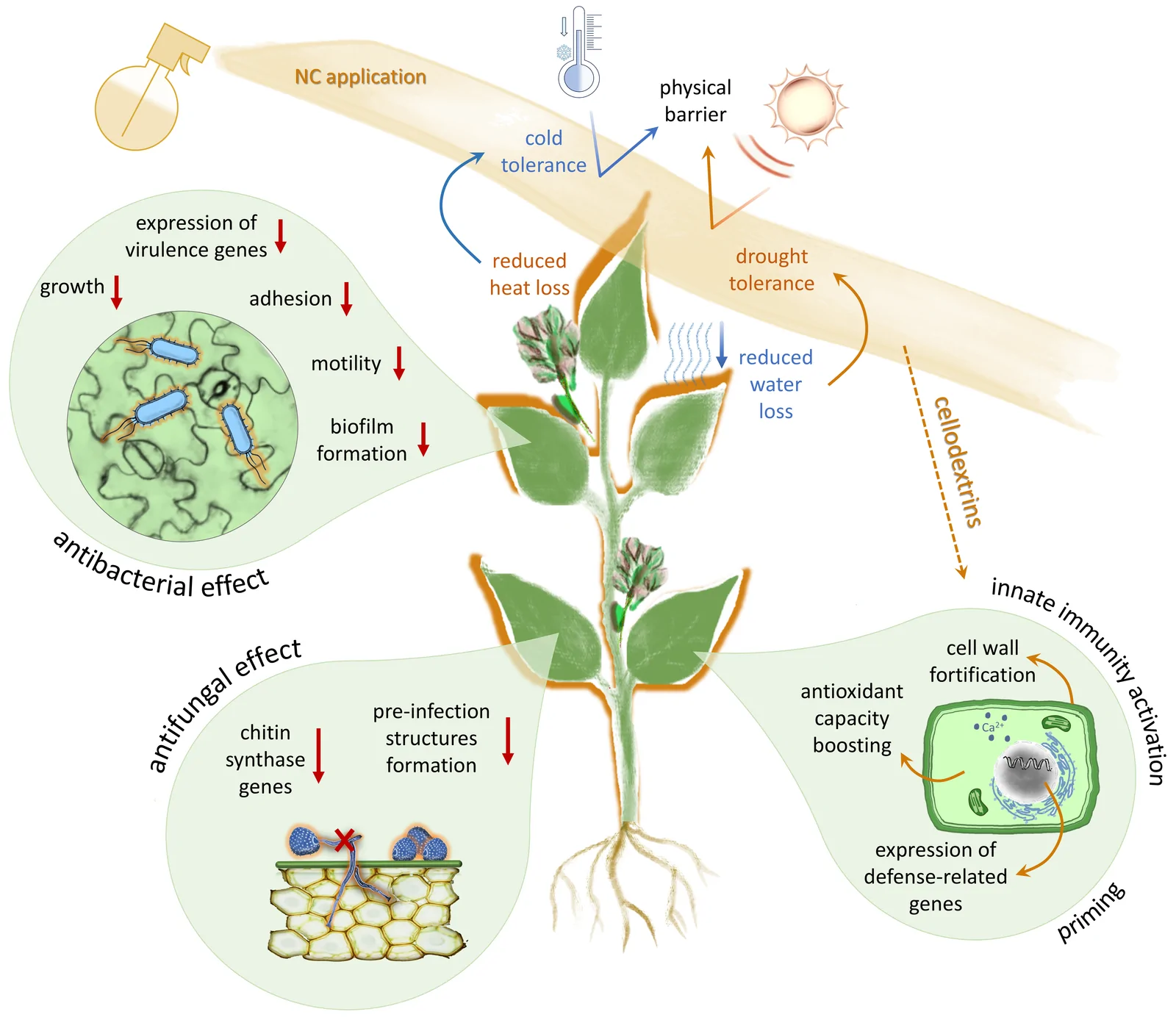
NC effects on plant and putative mechanisms of NC-induced plant stress adaptation. Dark-red arrows reflect downregulation or suppression; the dark-red cross designates prevention of germ tube and appressoria formation.

The basis of CNC- and CNF-mediated beneficial effects on plant stress tolerance can be divided into two main categories: a physical barrier against environmental factors and the induction of intrinsic plant responses that lead to stress adaptation. This dual mode of action is schematically illustrated in Figure 2. CNC-enhanced plant tolerance to cold and drought stress is mainly based on the formation of a “shield”: a CNC film serves as an insulator that preserves plant tissues from excessive water loss and low-temperature damage. However, numerous physiological and metabolic changes observed in CNC-treated plants indicate that this nanomaterial induces particular biochemical processes, activating cascades linked to stress adaptation [64]. Thus, the synergistic effect of physical and physiological responses underlies CNC-enhanced plant tolerance to abiotic stresses. Moreover, similar to the abovementioned stresses, salt and heat stresses are associated with dehydration, osmotic changes and ROS accumulation, all of which have highly adverse effects on plant growth and productivity. The cascades of reactions triggered by salt, heat and drought stresses are highly overlapping [58,60]. Thus, it can be assumed that treating plants with CNC formulations would also have positive effects on plant fitness by reducing oxidative stress and inducing transcriptional and post-transcriptional changes, leading to enhanced stress tolerance. However, these aspects remain to be further explored in future studies.

The mechanisms underlying the ability of CNC to reduce epiphytic bacterial survival and its effects on *P. syringae* growth and biofilm formation are far from clear. In earlier studies, CNC were shown to hinder *P. fluorescens* adhesion to solid surfaces [80], to prevent biofilm formation and to compromise the integrity of model lipid membranous vesicles and bacterial cells [81,82]. Sun et al. also reported that 0.1% CNC induced depletion aggregation of *P. aeruginosa* cells [83]. However, Schiavi et al. did not reveal any damaged *Pto* cells after incubation with CNC but observed increased bacterial aggregation and reduced motility [67], which is in line with the results obtained by Sakata et al. [70], who also demonstrated impaired bacterial movement on leaves treated with CNF. Squire et al. [68] showed the absence of any direct negative effect of 0.05% CNC on *P. syringae* growth in liquid medium during an 80 h OD monitoring study.

Several studies suggest that CNC act as elicitors that enhance plants’ ability to respond and adapt to various biotic and abiotic stresses. This hypothesis is based on biochemical [64] and transcriptomic studies [64,68]. The upregulation of numerous stress-responsive genes was observed upon both long-term (20 days) treatment of *F. arundinacea* plants [64] and short-term (1 h) treatment of *A. thaliana* seedlings [68]. Gene Ontology enrichment analysis revealed that upregulated genes included those related to oxidative stress response, CW fortification, and bacterial/fungal pathogen invasion. Moreover, in *F. arundinacea* plants, CNC application led to the upregulation of genes associated with lignin synthesis and photosynthesis [64].

It can be hypothesized that CNC are not likely elicitors themselves; however, some CNC preparations, due to their production technology, which includes acid hydrolysis, contain cellooligosaccharides that act as pathogen-/damage-associated molecular patterns (PAMPs and DAMPs) and potent elicitors of the plant immune response [84,85]. In addition, CNF preparations obtained by acidic hydrolysis and TEMPO-oxidation also contain cellooligosaccharides that serve as elicitors [85]. Indeed, there is substantial evidence for the biological activities of cellobiose and cellodextrins as elicitors [86,87,88,89,90]. Cellodextrins have been reported to induce ROS generation, cytosolic calcium influx, activation of MAPK cascades, and expression of genes associated with the defense response. Apart from eliciting a general immune response, cellooligomers upregulate genes involved in CW integrity maintenance. Therefore, the CNC priming effect might be explained by the presence of cellooligosaccharides rather than by intrinsic CNC properties. Studies on the antibacterial and antifungal activity of CNF indirectly support this hypothesis. In contrast to CNC, all CNF preparations (Nanoforest^®^) discussed in Section 3 are obtained by mechanical water cleavage (aqueous counter collision method), which completely excludes the formation of cellodextrins because the energy of the water jet is not intense enough to break the covalent β-1,4-glycosidic bonds of the cellulose molecules but is only able to disrupt the hydrogen bonds that hold cellulose chains together [34]. Therefore, this specific type of CNF preparation lacks the cellooligosaccharides that act as elicitors of plant immunity. Indeed, studies investigating the effects of CNF on plant biochemistry and gene expression have demonstrated that their observed antibacterial and antifungal properties are directed at the pathogen rather than the plant. Specifically, the expression of pathogenesis-related and other defense-related genes, including those involved in the phenylpropanoid and isoflavonoid pathways for phytoalexin production, was not significantly upregulated in CNF-treated leaves compared to controls. In contrast, distinct effects were observed on the motility, adhesion, and gene expression of both fungal and bacterial pathogens [70,72]. The authors proposed that the CNF-mediated alteration of leaf surface wettability is the primary mechanism driving this reduction in pathogen propagation and plant colonization.

At the molecular level, this shift in surface wettability interferes with pathogen behavior. Virulence-related genes in *Pcal*, including those encoding type III effectors (which suppress PAMP-triggered immunity), coronatine biosynthesis (which induces stomatal reopening), and flagellin (essential for motility), were significantly downregulated on CNF-treated leaf surfaces. This impairs bacterial motility and compromises the pathogen’s ability to suppress plant immunity and reopen stomata, decreasing its overall colonization capacity [70,76]. Similarly, in the fungal pathogen *P. pachyrhizi*, genes involved in chitin synthesis were significantly downregulated, leading to reduced appressoria formation and decreased infection efficiency on CNF-treated soybean leaves [72]. The authors linked this antifungal effect to the alteration in leaf surface hydrophobicity, suggesting that the activation of *P. pachyrhizi* genes associated with appressoria formation is highly dependent on the fungus “sensing” a hydrophobic surface. This hypothesis aligns with previous findings highlighting the critical role of cuticular waxes and surface hydrophobicity in initiating infection by various fungal pathogens [72].

In summary, NC protects plants through a dual mechanism that combines physical barrier formation against environmental and biotic stressors with the activation of intrinsic molecular defense pathways. While CNF primarily act by altering surface properties to inhibit pathogen colonization, CNC-mediated protection arises from an interplay between their role as a physical protective shield and the elicitor activity of CNC preparations. This elicitor activity is largely driven by cellodextrins generated during CNC production, which prime plant immune responses. The combination of physical shielding and biochemical priming underlies the broad-spectrum efficacy of NC in improving crop fitness.

## 5. Challenges and Future Directions

Despite the promising laboratory results, the transition of NC from controlled environments to large-scale agricultural usage faces several significant hurdles. First, the current production costs of NC remain relatively high due to the energy-intensive nature of mechanical homogenization, the use of specific chemicals or enzymes, and the logistical challenges of biomass collection and processing [91,92,93]. Second, establishing product standardization for agricultural NC is highly challenging. The physicochemical properties of NC, such as particle morphology, size distribution, aspect ratio and charge, are highly dependent on the raw biomass source, extraction method, and specific process parameters. Even batches derived from the same raw material can exhibit variations [94]. Thus, the problem of consistency still remains unresolved: scaling up NC production necessitates overcoming difficulties related to batch-to-batch uniformity, as well as fast, reliable and precise methods of quality control at each production stage at an industrial scale. Additionally, storage and stability present practical challenges; for instance, aqueous NC suspensions are susceptible to microbial degradation over time, while drying processes can lead to irreversible agglomeration, complicating long-term storage, formulation, and transportation [95]. Third, although the final NC product is “green”, conventional extraction processes (e.g., harsh chemical treatments required for lignin removal or acid hydrolysis) can be resource-, energy-, and chemical-intensive [10,16]. Developing truly “greener,” low-energy manufacturing methods and closed-loop purification systems is therefore essential to ensure the overall sustainability of the production pipeline.

While it is generally accepted that NC is a biocompatible and rapidly biodegradable alternative to synthetic agrochemicals, its environmental and safety profiles should be accurately considered. Pure CNC and CNF exhibit negligible phytotoxicity at agricultural application rates. However, their biological impact can be influenced by production residuals, leading to altered toxicity profiles of the final preparations. Furthermore, while rapid biodegradation prevents long-term environmental accumulation, optimizing application dosages remains critical to avoid potential physical effects (e.g., stomatal clogging) at excessively high concentrations.

Despite these challenges, the beneficial properties of NC position it as a highly promising material for the future of agriculture. Owing to their plant-based origin, CNC and CNF are widely recognized for their biosafety and nontoxicity. As renewable resources, they align perfectly with the principles of a circular bioeconomy, valorizing agricultural and forestry biomass waste into high-value products. This sustainability offers a pathway toward “greener” agriculture by significantly reducing the use of traditional agrochemicals.

Taking into account that plant treatment with CNC modulates and reduces oxidative stress, CNC-based preparations hold significant potential to enhance plant tolerance to a variety of abiotic stresses, including drought, salinity, cold and heat, all of which typically trigger a ROS burst [47,55,56,58]. Moreover, as CNC films protect plant tissues from low temperatures and excessive water loss, they could potentially be used to protect plants from heat stress according to a similar mechanism of action, functioning as a physical barrier and a molecular inducer that affects the expression of numerous genes involved in adaptation to abiotic stresses.

Moreover, NC could be regarded as a promising tool for plant pathogen control because of its unique mode of action based mainly on physical properties. Due to this, NC functioning as a “shield” and creating unfavorable conditions for pathogen entry [66,70,72,73] would help overcome the growing problem of fungicide and antibiotic resistance.

Based on the physicochemical properties discussed in this review, we propose several putative ways for the future utilization of NC in plant protection. Given its film-forming properties, we hypothesize that NC could potentially serve as a physical “trap” for spores and pathogenic microorganisms, hindering their penetration into plant tissues and the initiation of infection. Furthermore, these characteristics of NC lay a conceptual foundation for developing novel strategies for plant antiviral protection. For example, it could be suggested that NC-derived films or NC-based gels may serve as mechanical barriers that prevent virus transmission by insect vectors (e.g., aphids). Similarly, we believe that NC-containing gels supplemented with insecticides could be developed to protect crop plants against sap-feeding insects and, therefore, insect-transmitted viruses. Finally, CNC-mediated activation of plant innate immunity enhances general antiviral defense. Thus, NC could be utilized as a versatile formulation with antifungal, antibacterial, and potentially antiviral action, covering a wide range of pathogens.

Importantly, NC is already moving from the lab to real field applications. For instance, sprayable CNC suspensions invented by researchers from Washington State University and, in parallel, by Korean scientists are currently undergoing field trials in commercial orchard settings to protect flower buds of fruit trees (e.g., apples, cherries, peaches) from devastating spring frost damage, successfully acting as an insulating physical barrier. Similarly, recent commercial initiatives, such as those advanced by Marubeni Corporation, have begun field trials of CNF-based agricultural sprays, specifically designed to confer antibacterial and antifungal protection to crops. These developments demonstrate the practical viability of NC, bridging the gap between laboratory innovation and field-scale reality and paving the way for its widespread adoption in sustainable crop management.

## 6. Concluding Remarks

NC is generally regarded as a promising material for agriculture with numerous application methods. In most studies, NC has played an auxiliary role, being used as a structural component of nanocomposites for the delivery and controlled release of nutrients, pesticides and growth stimulators. However, the intrinsic biological activity of pure plant-derived NC has increasingly come into focus. Upon closer examination, it has become apparent that treating plants with CNC induces a general immune response, activating genes involved in reducing oxidative stress, CW fortification and antibacterial/antifungal defense. However, this elicitor activity seems to be largely driven by cellulosic oligosaccharides generated during processing, which act as DAMPs. Notably, this CNC-induced elicitation does not lead to a growth–immunity trade-off; it activates defense mechanisms without negative effects on plant biomass accumulation or development, a critical advantage over many synthetic chemical elicitors. Furthermore, CNC provide protection against abiotic stresses, such as cold and drought, by acting as both a physical insulating barrier and a molecular modulator.

In the realm of biotic stress, CNC demonstrate a strong capacity to inhibit foliar epiphytic bacterial growth and prevent biofilm formation, allowing them to be utilized as agents to control plant infection by bacterial pathogens. CNF have been shown to form coatings that convert the leaf surface to hydrophilic, leading to suppressed colonization of plant tissues by pathogenic bacteria and fungi. Together, these mechanisms enable NC to protect crops against a wide spectrum of pathogens.

Although NC satisfies crucial ecological requirements, such as biodegradability and minimal toxicity to ecosystems, its large-scale agricultural application faces significant hurdles. Overcoming these barriers requires minimizing production costs and simultaneously transitioning away from conventional resource-, energy-, and cost-intensive cellulose extraction that involves harsh and non-eco-friendly processes. Developing “greener” manufacturing methods is therefore essential. Furthermore, scaling up NC production necessitates overcoming challenges related to batch-to-batch uniformity and precise control of oligosaccharide profiles to ensure consistent elicitor activity. Cost-effectiveness, sustainable low-energy manufacturing, and strict reproducibility will be the decisive factors in expanding the use of NC, both in its pure bioactive form and as an advanced carrier for agrochemicals. If these technical and economic challenges are overcome, NC is poised to become a transformative alternative to traditional agrochemicals. Derived from renewable resources, biodegradable, and biosafe, it offers a green, highly effective pathway to ensure reliable harvests and drive the transition toward eco-friendly agriculture.

## Data Availability

No new data were created or analyzed in this study. Data sharing is not applicable to this article.

## Abbreviations

The following abbreviations are used in this manuscript:

CNC	cellulose nanocrystals
CNF	cellulose nanofibers
CW	cell wall
DAMP	damage-associated molecular pattern
NC	nanocellulose
PAMP	pathogen-associated molecular pattern
ROS	reactive oxygen species
